# Revisiting Paraphrenia: A Case Report

**DOI:** 10.7759/cureus.34391

**Published:** 2023-01-30

**Authors:** Filipa Andrade, Cláudia Reis, Márcia Mota, Henrique Salgado

**Affiliations:** 1 Psychiatry, Centro Hospitalar Universitário de São João, Porto, PRT

**Keywords:** psychotic disorder not otherwise specified, atypical psychosis, delusional disorder, paranoid schizophrenia, paraphrenia

## Abstract

Paraphrenia is a chronic psychotic disorder characterized by a strong delusional component with preservation of thought and personality. It was first introduced as a disorder associated with paranoid dementia and paranoia, but with less personality deterioration than schizophrenia and without fulfilling the clinical features of a delusional disorder. This classic diagnostic entity is not currently listed in main diagnostic systems, rendering delusional disorders difficult to classify in cases that resemble the concept of paraphrenia. We revisit the concept of paraphrenia through a critical review based on a clinical vignette of a patient followed at the psychiatry department of the University Hospital Center of São João.

## Introduction

Paraphrenia refers to a chronic psychotic disorder characterized by a strong delusional component with preservation of thought and personality. It is a classic diagnostic entity not currently listed in main diagnostic systems, rendering delusional disorders difficult to classify in some cases that resemble the concept of paraphrenia. The prevalence rate is unknown, but it is suggested that paraphrenia occurs only one-tenth as often in an inpatient population as does schizophrenia [1,2].

Kraepelin (1913) proposed the term paraphrenia to define a chronic psychotic disorder similar to dementia praecox, with a strong delusional component, but with better-preserved affects and without disturbances of volition [3]. A few years after Kraepelin's description, Mayer-Gross published a report concluding that patients diagnosed with paraphrenia gradually progressed to a different diagnosis, suggesting that the distinction between paraphrenia and schizophrenia was unfounded [1]. Munro (1991) recognized paraphrenia as a distinct clinical entity, referring to patients who presented with a delusional disorder but maintained affective rapport and lacked formal thought deterioration and grossly disorganized behavior [1,4].

In 1999, paraphrenia´s diagnostic criteria were redefined [1]. According to the authors, patients who presented with delusional paranoia without a well-encapsulated delusional system and who didn’t appear to have a profound disturbance of thought and personality could be diagnosed with paraphrenia [1]. Additionally, they considered that the disease typically began in middle or advanced age, although admitted that there may be cases in the third decade of life [5]. Although it has a chronic course, the evidence shows better short-term response rates with antipsychotic treatment when compared with paranoid schizophrenia [6-8]. Although the clinical outcome seemed to be satisfactory, delusions often prevent good compliance with treatment [4,8]. Cluster A personality disorders, sensory deficits (especially auditory), migrant status, and other significant stressful events are considered risk factors, with no significant link with a family history of schizophrenia or psychosis [6,9,10]. The identification of these clinical features allowed the distinction of paraphrenia as a psychotic disorder with a specific nosographic autonomy.

Since the publication of Mayer-Gross’s report, the discussions on paraphrenia lessened and paraphrenia was excluded from the International Classification of Diseases (ICD) and Diagnostic and Statistical Manual of Mental Disorders (DSM_ series. However, the question of whether paraphrenia is a distinct diagnostic entity remains.

## Case presentation

A 54-year-old man, a married factory worker, was assessed in an emergency department for presenting persecutory delusions, auditory hallucinations, and changes in behavior in the prior four weeks. He believed his neighbors had installed cameras in his house to follow him everywhere, for which he adopted some protective behaviors, such as hiding his genitals while having a bath and staying awake at night in case the neighbor entered. Furthermore, he had auditory hallucinations in the form of commenting voices and insults. There was no formal thought disorder. He was anxious, with a depressed mood, and having great difficulty falling asleep. No relevant medical history and no relevant personal or family psychiatric history were noted.

He was submitted to medical investigation in order to exclude possible organic causes of the symptoms. His toxicology screening was negative and no organic brain damage was observed. There were no changes reported in the EEG and CT scans. A full blood count showed normal values, and no abnormalities were found in his renal (creatinine, urea, and glomerular filtration rate), liver function, electrolytes, and C-reactive protein. Normal values were found for thyroid-stimulating hormone, free thyroxine, and haptoglobin. Also, viral markers for human immunodeficiency virus 1 and 2, hepatitis C virus, and hepatitis B virus were negative.

Three months later, the patient attempted suicide in an act of despair. The anguish associated with the presence of psychotic symptoms led to the ingestion of poison and subsequent hospitalization. Although he had no insight into his clinical condition, he showed compliance with the medication but no symptom improvement. Maintenance of functionality at work was reported. Whilst hospitalized the patient’s symptoms evolved favorably, with an improvement in psychotic symptoms. Aripiprazole 10 mg was prescribed. He was diagnosed with “psychosis not otherwise specified” and was regularly followed in outpatient consultation after discharge.

In the following months, the patient complained of insomnia and persecutory delusions and the hallucinations resurfaced. No other symptoms were identified. The patient reported therapeutic compliance. Despite multiple therapeutic adjustments, the symptoms remained and he was admitted for day hospital care due to treatment refractoriness. Clozapine 350mg was prescribed, with improvement. A neuropsychological assessment was carried out, which did not reveal cognitive deterioration. He maintained the diagnosis of “psychosis not otherwise specified” and was regularly followed in consultation. During the observation period, the patient showed no personality deterioration and cognitive function was preserved. The patient revealed less preoccupation with delusions and hallucinations. Despite being able to retain interpersonal functioning, the patient did not resume work. Marital functioning declined since the beginning of the illness.

## Discussion

In this clinical case, the patient had a chronic psychotic disorder with non-encapsulated persecutory delusions, accompanied by auditory verbal hallucinations. The condition had been present for approximately 10 months, during which the patient had shown personality preservation, retaining interpersonal functioning, with appropriate affect and no cognitive deterioration.

The disorder began in middle age, in a patient with poor education and without any family psychiatric history and no evidence of premorbid maladjustment. The first symptoms appeared after a period of significant stressful events, considered a putative risk factor for paraphrenia [6,11]. These clinical features support the diagnosis of paraphrenia as proposed by Ravindran, Yatham, and Munro (Table 1).

**Table 1:** Diagnostic criteria for paraphrenia redefined Source: [1]

«A delusional disorder of at least 6 months duration, characterized by the following:
1. Preoccupation with one or more semi-systematized delusions often accompanied by auditory hallucinations. These delusions are not encapsulated from the rest of the personality as in delusional disorder.
2. Affect is notably well-preserved and appropriate. Even in acute phases, there is an ability to maintain rapport with the interviewer.
3. None of the following: intellectual deterioration, visual hallucinations, incoherence, flat or grossly inappropriate affect, or grossly disorganized behavior at the times other than during the acute episode.
4. Disturbance of behavior is understandable in relation to the content of delusions and hallucinations.
5. Only partially meets DSM-IV Criterion A for schizophrenia and no significant organic brain disorder.»

The patient’s mood-related symptoms seem to be secondary to psychotic symptomatology, as they improved with antipsychotic treatment and the improvement of psychotic symptoms. Thus, the diagnosis of mood disorder or schizoaffective disorder is unlikely. There was also a scarcity of negative symptoms and no formal thought disorder.

No disturbance of volition was observed, and the patient showed no personality deterioration. Cognitive and social functioning were preserved. The patient’s behavior was organized and understandable in light of the delusional and hallucinatory content. Thus, schizophrenia and schizophreniform disorder are less likely diagnoses. The non-encapsulated persecutory delusions and the prominent hallucinations render delusional disorder less likely. In addition, the complementary diagnostic tests allowed the exclusion of organic pathology, excluding drug-induced psychosis, dementia, and other psychotic disorders due to general medical conditions or organic brain damage.

The paraphrenia clinical outcome is often satisfactory, with intellectual functioning, daily living, working activity, and social relation generally unimpaired [12]. Despite its chronic and relapsing course, paraphrenia appears to have a good response to antipsychotic medication [1,12]. In the case reported here, the patient’s initial improvement was later followed by relapse with resistance to standard neuroleptics, responding only to clozapine. The patient has been able to retain interpersonal functioning and an adjustment to the real world but hasn’t returned to work and marital functioning deteriorated. In spite of its satisfactory outcome, many studies indicate that paraphrenia is longitudinally associated with a degree of cognitive impairment [7,11,13,14]. Also, social outcomes can decrease with advancing age, given the age-related difficulties and habitual isolation of the elderly, making rehabilitation harder [7]. As such, close patient follow-up is warranted in order to identify relapse, new symptoms onset, and recovery needs.

## Conclusions

In clinical practice, there are cases of paranoia without the well-encapsulated delusional characteristic of a delusional disorder, yet with less personality deterioration and without thought disturbance observed in paranoid schizophrenia. In recent years, only a few systematic studies on paraphrenia are available, leaving the disorder on a spectrum between delusional disorder and paranoid schizophrenia. In the light of the current diagnostic systems, the case reported may be labeled as “atypical psychosis”, “delusional disorder”, or “psychotic disorder not otherwise specified” in the absence of a better diagnostic category. Re-establishing paraphrenia as a viable diagnostic entity could allow more accurate diagnosis and further research could benefit patient care.
